# Verification of prostate treatment setup using computed radiography for portal imaging

**DOI:** 10.1120/jacmp.v3i2.2582

**Published:** 2002-03-01

**Authors:** Richard Whittington, Peter Bloch, Della Hutchinson, Bengt E. Bjarngard

**Affiliations:** ^1^ Radiation Oncology Department School of Medicine University of Pennsylvania 3400 Spruce Street Philadelphia Pennsylvania 19104

**Keywords:** radiation therapy, portal imaging, computed radiography, digital image processing

## Abstract

A non‐film‐based system was used to obtain high quality portal film images. Digital portal images were obtained with a computed radiography (CR) system, in which the film is replaced with a photostimulable phosphor plate. Digital processing of portal images enhanced the display contrast using regional histogram equalization. The images were compared to images on radiographic film, exposed in the same cassette. The contrast‐enhanced CR images of prostate treatment fields facilitated identification of the entire contour of the ischium, the location of the pubic symphysis, and the ischial tuberosity to determine the anterior and inferior locations of the prostate and bladder. Identifying the coccyx on the processed portal images permits the physician to locate accurately the posterior wall of the rectum. In each case the quality of the CR image was judged by the clinician to be superior to conventional portal film. The identification of these anatomical structures on the portal images is clinically important for verifying 3D conformal therapy of the prostate. With the same CR system one may acquire digital treatment portal and simulation images. This provides a foundation for a picture archival communication system for radiation oncology. Existing software can be used to register these digital portal and simulation images to facilitate verification of treatment setup.

PACS number(s): 87.53.–j, 87.57. –s

## INTRODUCTION

Recent advances in radiation oncology, including the development of computerized tomography (CT) simulators, three‐dimensional treatment planning systems, stereotactic frames, and intensity‐modulated radiation permit the use of more complex, noncoplanar methods to treat tumors with higher doses and tighter margins. This is intended to increase local control in the treatment of a number of tumors. These developments have already affected the treatment of prostate cancer, intracranial tumors, and hepatico‐biliary tumors.^1^ ^–^ ^3^ Three‐dimensional conformal radiation therapy has increased the precision of treatment delivery methods. However, verification of the treatment set‐up fields is more complicated requiring beams‐eye view projections of the internal anatomy that are reconstructed from axial CT studies. The reduced planning‐target volumes required to spare normal tissues and the tighter definition of tumor margins makes the accuracy of the treatment setup more critical.^4^

For the evaluation of coplanar field treatments two anatomical points on at least two images are used to determine the accuracy of a coplanar field arrangement. With noncoplanar fields identification of three anatomical reference points in each portal image is used to confirm the accuracy of the setup. The identification of these points is complicated by the very limited contrast between bone and soft tissues in radiographic images acquired with high‐energy photon beams. Digitally acquired portal images can be computer processed to enhance the displayed contrast in portal images. This was investigated in this study, and the ease in identification of anatomical landmarks in these processed images was assessed by clinicians. The clinical impact of computer image processing was evaluated for lateral and small, oblique portal fields used for three‐dimensional (3D) conformal treatment of the prostate because in these treatment fields anatomical structures are difficult to identify on conventional portal images.

Computed radiography (CR) was used to acquire digital portal images.^5^ ^,^ ^6^ In CR, the radiation oncology technologist substitutes a photostimulable phosphor plate (PSP) for the film in the metal‐screen cassette. CR systems^7^ ^–^ ^10^ are used extensively in diagnostic radiology to provide digital images for picture archival communications systems (PACs). The radiologist retrieves the image data file from the computer archive and makes the diagnosis from the image displayed on a monitor. The radiation oncologist could do the same with potential advantages. The digital portal images could be computer processed to increase the display contrast between bone and soft tissue. This overcomes a major limitation of the low tissue contrast available on conventional radiographic portal films. The enhanced display contrast of the processed portal image helps the physician to identify with confidence multiple anatomical points in the image that can be used to verify the clinical setup. In addition, commercially available software for registering digital portal and simulation images would be useful for evaluating the treatment setup accuracy.

## METHODS

### A. Computed radiography scanner

The PSP (Agfa ADC plates) used with the CR system contains a phosphor (BaFBr:Eu) in an organic matrix coated on a flexible substrate approximately 1‐mm thick. When irradiated with x rays or electrons, the phosphor stores the absorbed energy in quasistable electronic states. When the plate is scanned with a red laser beam, the phosphor is stimulated to emit light whose intensity is proportional to the locally stored energy.^9^ ^,^ ^10^ The Lumisys Model ACR‐2000 CR system was used to scan the PSP. The scanning time is approximately 30 s. For acquiring diagnostic or simulator images, the PSP is scanned with a pixel size of 174 μm (2048 pixels/line). Image acquisition with the 347‐μm pixel size (1024 pixels/line) is used for portal images. The portal‐image file size is approximately 2.5 megabytes, which is stored in DICOM‐3 compliant format.

For portal‐image acquisition, the sensitivity of the Lumisys scanner was reduced from that typically employed for diagnostic CR radiographic studies by lowering the voltage on the photomultiplier tube (PMT) used to detect the photostimulable luminescence from the PSP. The log of the signal from the PMT is digitized using a 12 bit analog‐to‐digital converter. The digital output from the CR Lumisys system is thus proportional to the logarithm of the luminescence from the PSP.

### B. Sensitometric and Resolution Measurements

The portal cassette, containing the lead screens, was placed in a holder mounted to the gantry 155 cm from the source. This is the position of the cassette when used to obtain portal images. It was irradiated to different doses with a 6‐MV photon beam that had a field size of $4\times 4{\text{cm}}^{2}$ at isocenter. The PSP was scanned as described above. The DICOM output file from the scanner was read using the interactive data language (IDL) procedure READ_DICOM.^a^ The average detector response in a 1‐cm^2^ region in the center of the field was measured as a function of relative dose. In addition, the response of the CR system was measured with a plastic step wedge [see Figure 2(a)] placed at isocenter, 55 cm from the PSP detector.

**Figure 1:**
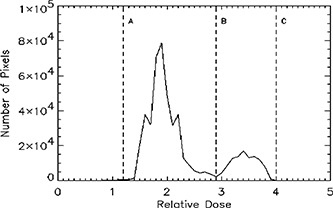
Number of pixels as a function of relative dose in prostate treatment portal image. Pixels with dose levels lying between *A* and *B* correspond to points under the collimator jaws or blocks. Pixels with relative dose between *B* and *C* correspond to points within the treatment field.

**Figure 2:**
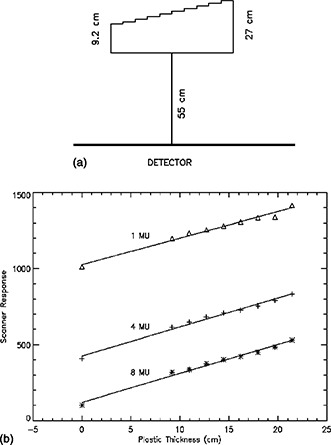
(a) Plastic step‐phantom and geometry used to measure the scanner response. (b) Dose response of CR system as a function of the set number of monitor units and absorber thickness.

The overall spatial resolution of a digital portal system can be expressed as the product of the modulation transfer functions (MTFs) of the various components of the imaging system. These

include the MTF's associated with (i) the target size and energy of the x‐ray source; (ii) the metal screens in the portal cassette; (iii) the distribution of secondary electrons produced in the metal‐screen; (iv) the film or PSP detector; and (v) the sampling aperture of the digitizer. We compared the MTF of the system with film and the PSP detector in the metal‐screen portal cassette. The MTF's of the PSP and film for portal imaging were calculated from measured edge spread functions (ESF). A 10‐cm thick lead block was positioned at isocenter on the portal cassette with its edge aligned with the centerline of the radiation field. The lead block on the metal‐screen cassette that contained both the film and PSP was irradiated with a 6‐MV photon beam. The image of the edge on film was scanned with a film microdensitometer (Lumisys Lumiscan Model 100) with the same scanning aperture or pixel size (347 μm) used to acquire the digital image on the PSP. The scanned images were corrected for the dose response of the CR and film systems, respectively. The derivative of the ESF measured on both the PSP and film was used to define the corresponding line spread functions (LSFs). The MTF was calculated by taking the Fourier transform of the LSF The source, metal‐cassette, and digitizing aperture used to obtain the ESF of both the film and PSP detectors were identical. Thus any differences in the derived MTF for the two imaging systems would be associated with differences in the spatial resolution of the PSP and film detectors.

### C. Portal image processing

Portal images may be viewed directly on the monitor of the CR system. However, the display‐setting options available with the unit were optimized for diagnostic images. Special software procedures were developed in IDL for displaying portal images. These included windowing and regional histogram equalization, using the IDL procedure HIST_EQUAL.

The portal image was cropped to include both the treatment field and regions under the collimator jaws or blocks. The signal in each pixel was converted to dose using the measured dose‐response of the CR system. A histogram of the relative dose in each pixel of the cropped image data file was calculated and used for selecting the window levels of the displayed image. Figure 1 shows, for example, the number of pixels at each dose level within the cropped portal image for an anterior prostate treatment field.

Pixels with higher x‐ray doses are in regions within the treatment field, whereas the lower dose regions correspond to areas under the collimator jaws or blocks. The window settings between B and C are used for viewing the treatment field, where C corresponds to the maximum dose to any pixel in the image file. Window levels between A and B are used for viewing the anatomy under the collimators or blocks, where A is the minimum dose in the data file (taken to be the dose where 99.9% of the pixels receive a greater dose). Level B is a dose greater than level A and less than level C having the minimum number of pixels in the histogram. The operator can modify these default settings if desired. The images within the treatment field and under the blocks are superimposed and displayed on the monitor. In portal images acquired either with radiographic film or CR, the anatomical details in the darker regions of the image are often not perceptible. Enhancement of these darker regions can be achieved by rescaling the original image so that the histogram of the pixel intensities in the enhanced image is forced to be more uniform. This procedure, known as histogram‐equalization, significantly enhances the display contrast of a portal image.^11^ The operator can view a regional‐histogram‐equalized image by combining the histogram‐equalized image of pixels within the treatment field (pixels with signal intensities between levels B and C) with the histogram‐equalized image of regions under the block or collimator (signal intensities between A and B).

### D. Evaluation of clinical CR portal images

Radiation oncologists evaluated 78 clinical CR and film portal images. Portal images were obtained at the anatomical treatment sites shown in Table I. Of special interest in this study was the application of CR portal images for verifying the radiation fields used for treatment of prostate disease. To verify the position of the fields used for delivering conformal radiation to the prostate requires that the clinician easily identify on the portal image the entire contour of the ischium, as well as the precise location of the pubic symphysis and the ischial tuberosity. These anatomical landmarks are required to determine accurately the location of the anterior and inferior structures in the field, the prostate and the bladder. The identification of the coccyx allows the precise localization of the posterior structure of concern, the rectum. This multipoint evaluation of the fields can be checked with a graduated reticule that may be used when the portal image is obtained, or the image may be superimposed on a simulation film.

**Table I:** Distribution of CR portal images studied by anatomical sites.

Anatomical sites	Pelvic/prostate	Chest, eso, lung, and scapula	Hand‐neck, and spine	Extremities and nose	Brain
# CR images	42	24	3	7	2

Verification of dose delivery in 3D conformal treatment of the prostate is of major clinical importance. Several single‐institution experiences indicate that it is possible to deliver higher doses of radiation to the prostate without an unacceptable increase in the toxicities associated with treatment.^3^ ^,^ ^12^ ^–^ ^14^ Preliminary results from one randomized trial have also shown that these higher doses are associated with a reduced risk of biochemical recurrence.^12^ The higher doses delivered to the target volume while sparing normal tissues requires tight margins, which makes verification of treatment delivery even more critical.

For treatment planning of prostate disease a series of axial CT or MR images are obtained for delineation of the gross tumor volume and critical organ structures. However, radiographs of the portal treatment fields are used for verification of the treatment. Noncoplanar techniques and nonorthogonal fields increase the difficulty for a physician to evaluate the accuracy of treatment delivery due to the unfamiliar views of the anatomy provided by these different projections. To overcome this difficulty, a beam's eye view of the anatomy is generated from axial CT images and used for comparing differences between the planned and delivered treatment fields seen on portal images.^15^ ^–^ ^17^ This allows an assessment of the accuracy of the treatment delivery, but it is still limited by the poor contrast resolution available with film portal imaging. CR acquired portal images were computer processed to enhance the contrast between bone and soft tissues. Physicians were asked to evaluate the use of the CR image for verification of the treatment setup, and to judge the ease in identifying anatomical structures in these images compared to the film images. Film and CR portal images were obtained at the same time by placing both a film (Sterling Cronex 4) and PSP in the cassette with lead screens. An opaque film (>4 optical density) was placed between the film and the PSP to minimize fogging of the film by prompt luminescence from the PSP. The cassette was placed in a holder attached to the gantry 155 cm from the source. To obtain a portal image, three to five monitor units were given to the treatment field and two to three additional monitor units with the collimator jaws opened. All the images were obtained with 6‐MV x‐ray beams. The physicians were asked to evaluate the CR image for verification of the treatment setup, and to judge how the image content compared to the film image.

## RESULTS

### A. Sensitometric properties and spatial resolution of CR system

The output from the Lumisys CR system is the logarithm of the signal from the PMT in the instrument. The pixel intensity, which corresponds to the signal (*I*) from the CR system was described by equation 1, where *m* and *b* are obtained using a least‐squares fit analysis. The pixel intensity or scanner response is expected to vary with the logarithm of the dose (*D*) to the detector, which is directly proportional to the number of monitor units (MU) used to expose the PSP. Figure 2 shows the scanner response (I) from the CR system measured for 1, 4, and 8 delivered monitor units with various thickness' of absorber in a 6‐MV photon beam using the geometry and plastic step wedge shown in Fig. 2(a). The unattenuated dose to the PSP is approximately 0.4 cGy/MU. The response of the CR system as a function of dose is described by the relationship

(1) $$I=m\ln(MU+MU_{0})+b,$$

where *b, MU* ^0^, and m are 1433, 0.0021, and –431.9, respectively for our CR system. The energy fluence reaching the detector decreases nearly exponentially with absorber thickness, and since the output from the CR unit varies logarithmically with the dose, a linear variation of output with absorber thickness is observed.

**Figure 3:**
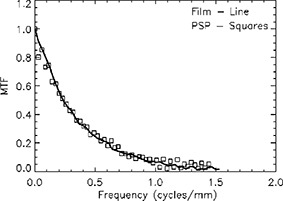
Modulation transfer function of PSP and film in a metal‐screen cassette.

Figure 3 shows the MTF for the film and CR system derived from the measured edge spread function. The maximum spatial resolution (1.44 cycles/mm) corresponds to the Nyquist frequency for the pixel size (347 μm) used for scanning. The reduction in the MTF below the Nyquist frequency is due to the angular dispersion of the Compton‐generated electrons in the lead screens and PSP. The nearly identical MTF's for the film and PSP plate in the portal cassette with lead screens indicates that the spatial resolution is not compromised with the use of CR portal imaging.

### B. CR portal‐image evaluation

**Figure 4:**
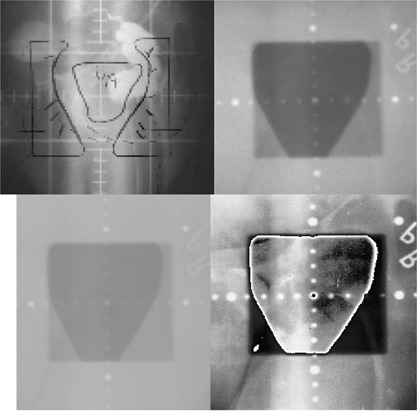
Portal images of an oblique prostate treatment field: simulation radiograph taken at 120 KVp (top left), portal film image of treatment field taken with a 6‐MV photon beam (top right), windowed CR portal image (bottom left), and windowed with regional histogram equalization (bottom right).

In these preliminary studies the radiation oncologists were satisfied that all the CR portal images could be used to verify the treatment setup. The subjective quality of the CR portal images was always the same or superior to images obtained with film. The CR image content for oblique and lateral pelvic treatment fields, following regional histogram equalization, was judged to be superior to the portal images acquired on film. A typical portal image obtained with film of a prostate lateral treatment is shown in Fig. 4 (top right). Figure 4 (top left) shows the simulator radiographic image of the treatment field with the block fields and planning target volume (mirror image of PTV) designated on the film. The portal images obtained using CR before and after regional histogram‐equalization are shown in Figs. 4 (bottom left) and (bottom right), respectively. In the histogram‐equalized image the ischia, pubic rami, and symphysis as well as the femoral heads are readily identified, whereas they are hardly perceptible on the portal film. Figure 4 (bottom right) shows the relative ease in defining the entire ischium and the lower sacrum and coccyx on a CR portal image of an oblique field used for 3D conformal treatment of the prostate. On anterior films, the usual reference points are the ischia, pubic rami, symphysis pubis, and femoral heads. On oblique films the acetabulum, ischium, and coccyx were usually used to evaluate the accuracy of the treatment portal setup.

## DISCUSSION

CR is an established method to produce digital diagnostic radiographs in many radiology departments. Since it has been shown in this and other studies^5^ ^,^ ^6^ that CR may also be used to acquire portal images for verification of treatment setup, one may conclude that digital acquisition of treatment portal images makes it possible for a radiation oncology department to establish a PACs that incorporates both treatment planning and setup verification images.

Both the CR and film portal images were digitized with the same size aperture (347 μm); thus the Nyquist frequency or spatial resolution limit for both systems are the same. Figure 3 indicates that the spatial resolution of the portal system with film or a PSP detector in the metal‐screen cassette are nearly equivalent. Thus using the PSP instead of film in the portal cassette does not significantly degrade the image performance. In addition, the inherent wide exposure latitude of the PSP detector permits it to be used for diagnostic imaging studies requiring low x‐ray exposures (typically less than 0.05cGy) for treatment set‐up fields in radiotherapy (with several cGy) and treatment verification images during radiotherapy delivery with 10–100 cGy. The gain on the photomultiplier tube may, however, need to be adjusted to avoid saturating the electronics. This wide exposure latitude makes CR a good system to be used for verification of intensity modulated radiotherapy.

The enhancement of the display contrast of anatomical structures on portal radiographs has been shown to decrease the time and improve the readings of a portal image.^18^ Acquiring digitized portal images, using either real‐time electronic portal imaging systems or CR, permits computer processing to be readily used to enhance the display contrast of anatomical structures both inside and outside the treatment field. For example, a comparison of the panels in Fig. 4 illustrates the enhancement in the display contrast that can be readily achieved with regional histogram equalization of a portal image. Without the computer processing the clinician could not identify the anatomical structures in the portal image required to adequately evaluate the setup of lateral or oblique prostate treatment fields. Using receiver operator characteristics (ROC) analysis Gur *et al*.^5^ have shown that radiation oncologists rated CR head‐and‐neck and chest portal images superior to conventional film images. However, they reported that the improvement in observer performance in reading CR portal images of pelvic treatment fields was not as great. In their study the graphic computer processing consisted in windowing and edge enhancement, but did not include regional histogram equalization. The preliminary findings of this paper suggesting improvement in identification of anatomical structures in prostate treatment field images following histogram equalization requires validation with a ROC imaging study.

We found that the contrast of a processed portal image using regional histogram equalization often results in an image with tissue contrast that more closely approximates the diagnostic image obtained at the x‐ray energies used to obtain radiographs at treatment simulation.

For the low‐energy x rays used in diagnostic radiology the difference in the photoelectric interactions results in a high radiographic contrast between bone and soft tissues. However, for the high‐energy x‐ray beams used for portal imaging in radiotherapy Compton interactions predominate resulting in much lower radiographic contrast between bone and soft tissues. The results presented demonstrate the value of having a variety of digital imaging processing tools available^19^ ^,^ ^20^ when reviewing portal images obtained from different anatomical sites.

## CONCLUSION

The images obtained of the treatment portal fields are important for verification of both the precision and accuracy of treatment delivery. The image contrast of conventional portal films is often very low, making it difficult for the physician to identify anatomical landmarks in the image. The display‐contrast enhancement that can be achieved with digital processing of portal films and real‐time electronic portal‐imaging devices (EPID) has been demonstrated in numerous studies.^4^ However, to use EPIDs routinely in the clinic is not a simple matter. The gain in image quality may not be sufficient to justify the expense and the logistics problems EPIDs create in a large and busy clinic. CR, on the other hand, can be used to acquire images with wide exposure latitude, spatial resolution equivalent to that obtained with portal films and low noise. It was previously demonstrated that computer enhanced contrast of portal film images^21^ and CR portal images^5^ significantly improved visualization of anatomical structures in portal images. The present study, however, did not make a comparison between histogram‐equalized CR and digitized portal film images.

A desktop CR unit costing less than $100 k can serve several treatment accelerators and simulators, thus controlling cost for the department. Using CR to acquire portal images would allow the radiation oncology community to take advantage of computerized image‐processing techniques and PAC technology with minimal expenditure of time and money. In addition, using existing software packages, e.g., portal imaging processing system (PIPSpro), the CR portal image may be registered with DRR and/or digitally acquired (using the same CR system) simulator radiographic images. Thus, one could integrate all the imaging studies required to evaluate the tumor coverage and to confirm the accuracy of the treatment setup.

## Footnotes

a Interactive Data Language Research Systems Inc., Version 5.2 (1998).
